# Is the NIH policy for sharing GWAS data running the risk of being counterproductive?

**DOI:** 10.1186/2041-2223-1-3

**Published:** 2010-09-01

**Authors:** Michael Krawczak, Jürgen W Goebel, David N Cooper

**Affiliations:** 1 Institut für Medizinische Informatik und Statistik, Christian-Albrechts-Universität zu Kiel, Arnold-Heller-Straße 3, Haus 31, 2105 Kiel, Germany; 2 Rechtsanwälte Goebel & Scheller, Schöne Aussicht 30, 61348 Bad Homburg v.d.H., Germany; 3 Institute of Medical Genetics, School of Medicine, Cardiff University, Heath Park, Cardiff CF14 4XN, UK

## Abstract

Through their current policy on data sharing, the National Institutes of Health (NIH) are inadvertently placing a serious and potentially insuperable burden upon non-US researchers who perform patient-based genomics studies in collaboration with US institutions. Because this policy could adversely affect future transnational scientific collaborations, we explore some of its likely consequences and suggest possible courses of remedial action wherever feasible.

## The NIH policy for sharing GWAS data

In January 2008, the US National Institutes of Health (NIH) put into effect their *Policy for Sharing of Data Obtained in NIH Supported or Conducted Genome-Wide Association Studies (GWAS) *(NOT-OD-07-088) [1]. The self-declared goal of this policy was 'to advance science for the benefit of the public through the creation of a centralized NIH GWAS data repository' (NOT-OD-07-088) [1]. The policy, which was revised and expanded in October 2009 so as to include DNA sequence data (NOT-HG-10-006) [2], was inspired by the view espoused by the NIH 'that the full value of GWAS to the public can be realized only if the genotype and phenotype datasets are made available as rapidly as possible to a wide range of scientific investigators' (NOT-OD-07-088) [1]. Irrespective of whether this view is justified (for example, on the basis of previous experience or persuasive scientific evidence), its implementation in the context of public funding practice may well place a serious burden upon researchers performing GWAS or large-scale sequencing studies. This would appear to be particularly true with regard to those researchers who initiated and conducted their research projects outside of the NIH sphere of influence, and who have only latterly come under its aegis by virtue of their being involved in collaborations with at least one NIH-funded partner. Although the NIH initiated a public consultation process in 2006, no representations were taken from non-US sources, as far as we can see. This was a serious omission, because the lack of any centralized health service provider in the USA implies that US researchers might in time have to become at least partially dependent upon well-characterized patient cohorts from other countries.

## The situation with non-NIH funded data

The major drawback of the current NIH policy is the mandatory requirement to place all genome-wide genotyping/nucleic acid sequence data (plus all relevant phenotypic information) from NIH-funded and NIH-supported studies into the NIH's own repository, the *Database of Genotypes and Phenotypes *(dbGaP) [3]. In this respect, the NIH is very clear about their position, stating in the *Implementation Guidance and Instructions for Applicants *for GWAS (NOT-OD-08-013) [4] that 'applications and proposals that include GWAS, regardless of the requested costs, are expected to include as part of the Research Plan either a plan for submission of GWAS data to the NIH-designated GWAS data repository, or an appropriate explanation for why submission to the repository will not be possible'. What explanation the NIH would regard as 'appropriate' here unfortunately remains unclear. This notwithstanding, we know from personal experience that the above requirement extends far beyond NIH-funded activities in the narrow sense so as to include non-NIH funded data from NIH-supported collaborative GWAS, irrespective of the proportion of funding actually provided by the NIH. The obligation to submit data to dbGaP applies equally well to situations in which only one component group is NIH-funded and where only a small proportion of the total cost of the study has been funded by the NIH.

## Some points of (constructive) criticism

In view of this overt interventionism with regard to academic working relationships, it seems worthwhile to explore some of the aspects of the NIH policy that could adversely (albeit wholly unintentionally) affect international collaborative efforts in patient-based genomics research. The NIH states that it 'will revisit and revise the policy and related practices as appropriate' (NOT-OD-07-088) [1]. We interpret this statement as an invitation to comment, in the spirit of constructive criticism, and to evaluate whether the NIH policy as currently formulated has unforeseen and/or unintended consequences for researchers. The following represents a list of difficulties potentially posed by this policy.

1. Without proper prior consent by their sample donors, no research institution would be in a position to submit individual-level genetic data to a publicly accessible database such as dbGaP. Because the scope of genomics research is inherently wide, and indeed often poorly defined at the outset, many research groups may well have been sufficiently prescient to have obtained broad consent from their donors, covering the possibility of transfer to third parties and the use of samples and data for general genetics research. However, unless the placing of data into international databases has been explicitly stated as an option in the information provided to donors at the outset, it cannot be assumed to have been automatically built into the study. Even though the NIH appears to have recognized this potential quandary by stating that they 'may give programmatic consideration to requests for funds or other resources needed to conduct additional participant consent when appropriate' (NOT-OD-07-088) [1], obtaining retrospective consent from a large GWAS cohort is likely to be difficult, if not wholly impractical, both in terms of the logistics required and the possible donor responses.

2. In August 2008, the NIH were obliged to change the access governance to dbGaP in response to a number of studies that convincingly demonstrated that individual genome-wide genotyping profiles could be identified even in aggregate datasets [5,6]. At first glance, the potential to re-identify individual anonymized contributors to a collection of single-nucleotide polymorphism (SNP) allele frequencies appeared to be implausible, even counterintuitive. Indeed, the marker-wise aggregation of genotypes in dbGaP into mere allele counts was initially regarded by the NIH as a sufficient means of anonymization. However, for each and every SNP, the minor and major allele frequencies in an aggregated dataset are necessarily shifted, almost imperceptibly but nevertheless consistently, towards the alleles of any target individual included in the dataset compared with a reference population not including that individual [5]. Although this would not be a problem in terms of re-identification when a hundred or even a thousand markers were involved [7], the combination of these biases over the hundreds of thousands of SNPs usually employed in a GWAS generates an effect that is very likely to become significant upon formal statistical testing, thereby rendering the individual in question potentially identifiable. Once this was realised, unrestricted access to the aggregated genotype data in dbGaP was promptly and appropriately abandoned, and all data were moved to a controlled area of the database. In the context of the present discussion, however, because these developments were pretty much unforeseeable even to genetics experts, the extent to which a lay person's consent to the inclusion of genetic data into a widely accessible database can be genuinely 'informed' seems to us scarcely tenable.

3. Despite the abandonment of unrestricted access to dbGaP, all aggregate and individual-level data in the NIH repository remain extremely sensitive, because of the inherent self-identifying property of genome-wide genotypes. Thus, a comparison between dbGaP and a reference genetic profile, generated for as few as 30 to 80 SNPs, would in practice allow the re-identification of any contributor [8], including restoration of linkage with phenotype data included in the database. As long as this identifiability problem remains unresolved, the sufficiency of the data-protection measures implemented by dbGaP will remain difficult to evaluate. It must not be forgotten that many, if not most, scientific collaborations involve partners with longstanding working relationships. Any violation of reciprocal agreements relating to the protection of shared data could result in a loss of trust between these partners. Such an outcome is likely to be a much greater deterrent to violation of privacy-protection requirements than the prospect of the imposition of unspecified sanctions by an anonymous database governance body.

4. At first glance, 'open consent', as promulgated for example by the Personal Genomes Project, may be one way around the identifiability problem [9]. If privacy were no longer to be promised to study participants, then there would clearly be no risk of breaking such a promise. However, widespread adoption of open consent in human genetics research may well be a slippery slope that would lead to the general erosion of the basic principles of informed consent in medical research. In a way, 'open consent' and 'informed consent' may even be regarded as mutually exclusive alternatives, because the act of a person giving their open consent implies the renunciation of any right to full information relating to the subsequent use of their sample(s) and associated data. Further, open consent may not only limit the group of potential participants in a given research project, but could also bias the results obtained.

5. During the consent process, the primary recipient of a sample or data item must be clearly recognizable by the sample or data donor. Otherwise, their consent cannot be assumed to be truly informed. Often, the donor's motivation to participate in a research project may be influenced by their tacit approval of, or long-term relationship with, a particular individual or a specific institution responsible for the project and potentially benefiting from it. If all genotype data are indeed destined to end up in a US-based international database under American curatorship, then all sample donors ought to be explicitly informed of this arrangement from the outset.

6. One of the key mechanisms by which the primary recipients of samples and data can ensure that the donor's consent is respected in collaborative research projects is by entering into bilateral, legally binding agreements with their project partner [10]. It is at best unclear to what extent non-US researchers would be willing (and indeed entitled) to delegate this responsibility to an anonymous Data Access Committee (DAC) under the auspices of the US authorities. In fact, neither NOT-OD-07-088 [1] nor NOT-HG-10-006 [2] makes any mention of transnational collaborations in which some of the participating institutions are legally bound by data-protection rules other than those of the USA. It is worth mentioning in this context that Article 25 of the EU Directive on Data Protection (95/46/EC) [11] requires that the transfer of personal data to a country outside the EU 'may take place only if [...] the country in question ensures an adequate level of protection'. As yet, the EU has not recognized the USA as belonging to the latter category of countries, largely because a regulatory framework comparable with the many national implementations of Directive 95/46/EC in the EU simply does not exist in the USA.

7. If the decision over who may or may not use a given set of GWAS data for whatever purpose is delegated exclusively to US authorities, this would to all intents and purposes undermine the authority of national governance bodies such as, for example, the UK's Human Genetics Commission [12], and local or regional ethics committees. Furthermore, although the NIH claims that their 'DACs will approve access only for research uses that are consistent with an individual's consent as defined by the submitting institution' (NOT-OD-07-088) [1], many requests to dbGaP can be expected to retain a certain amount of interpretational 'wiggle room' in this respect.

8. The actual scientific benefits to be derived from the free sharing of genetic research data are still unclear, and may eventually turn out to be negligible. The NIH confidently states that 'the potential for public benefit to be achieved through sharing GWAS data are significant' (NOT-OD-07-088) [1], yet they provide no concrete examples of such benefits, either actual or hypothetical. This notwithstanding, there are a number of methodological issues that actually place a serious question mark over the general utility of broad GWAS data sharing. Thus, ever-larger patient sample sizes may not automatically facilitate the reliable detection of ever smaller genetic effect sizes. Further, the hastily conceived combining of primary data from different sources could lead to ascertainment bias and population genetic differences that, if not taken into account at the data analysis stage, might actually increase the likelihood of false-positive results. Finally, because there are no generally agreed standards for the quality control of genotype or nucleotide sequence data, data heterogeneity may also become a serious problem that could only be alleviated if large amounts of accompanying technical information were also to be included in the data repository. Even then, however, the appropriate scientific use of this information would require methodological expertise that cannot be assumed to be available to the average user.

9. Without structured access to GWAS or nucleotide sequence data, meta-analyses of such data would also run the risk of becoming a problematic exercise, because the traditional commitment of primary data providers to certain collaborative consortia would no longer exist. Further, if the combined analysis of multiple data sets becomes feasible at the click of a mouse, it will become increasingly difficult for journal editors and peer reviewers to judge the scientific credibility of such efforts and to prioritize the publication of the results appropriately.

10. In addition to running the risk of impinging upon the basic tenets of academic freedom, a centrally imposed and rigidly enforced limit to the exclusive right of data exploitation by the primary data provider could be seen as both unfair and unjustified. In practical terms, the bottleneck in potentiating high-quality genetic epidemiological research is the recruitment and phenotyping of donors, not the generation of genotype data. This means that the major investment into a GWAS has often been made well before the genotype data become available, and by researchers who are not necessarily in a position to be able to exploit their data as rapidly as their US counterparts. In NOT-HG-10-006 [2], the NIH 'acknowledges the importance of recognizing the valuable and unique contributions made by the scientists who have collected the biological samples and associated phenotype information', yet nowhere is it made clear what form this recognition should take. For the original recipient of data and samples to benefit adequately from their work, a maximum embargo period of 12 months (as stipulated by the NIH) may simply be too short. It is difficult to understand why the NIH is unwilling to leave the decision as to when data are 'ready to go public' to the primary data providers themselves. It should be appreciated that unless there are very good reasons to the contrary, most genetic researchers working on a particular disease would normally be highly motivated to have their GWAS results replicated or otherwise improved by additional analyses, and hence may want to initiate the necessary collaborations themselves.

## Conclusions

The above considerations are not in any way intended to call into question the core motivation of the NIH policy, namely that 'maximizing the availability of resources facilitates research and enables medical science to better address the health needs of people based on their individual genetic information' (NOT-OD-07-088) [1]. On the contrary, any endeavour that helps to promote collaborative efforts in the field of patient-based genetics research is to be welcomed. In our view, however, the emphasis should be placed firmly on building a quality central resource to which the international scientific community would be glad to contribute data. In our own experience, scientists respond best to a variety of different forms of incentives rather than to edict or diktat. Given a relaxed regimen relating to data deposition, it is likely that researchers worldwide would opt to contribute their own GWAS and nucleotide sequence data to the NIH data depository on a voluntary basis, even if their work had not itself been NIH-funded. Conversely, the imposition of overly restrictive rules relating to data deposition could easily discourage non-US-based researchers from collaborating with NIH-funded groups, surely not the original aim of this well-intentioned yet still misguided NIH policy.

## Competing interests

The authors declare that they have no competing interests.

## Authors' contributions

All authors participated in the preparation of this work and read and approved the final manuscript.
